# Clinical Outcome Scores Post Medial Unicompartmental Knee Arthroplasty: A Comparison of the MAKO Robotic Arm versus the Oxford Conventional Approach

**DOI:** 10.5704/MOJ.2503.002

**Published:** 2025-03

**Authors:** CMP Tan, SSW Shih, V Ravichandra, ESH Quah, R Kunnasegaran

**Affiliations:** Department of Orthopaedic Surgery, Tan Tock Seng Hospital, Singapore

**Keywords:** unicompartmental knee arthroplasty, robot-assisted surgery, knee arthroplasty

## Abstract

**Introduction:**

Unicompartmental knee arthroplasty (UKA) has significant advantages over total knee arthroplasty (TKA). However, due to its need for precise positioning and soft tissue balancing, UKA failures and revision rates may be higher than that of TKA. Robotic-assisted UKA offers more accurate implant positioning, soft tissue balancing, improved lower limb alignment, and a reduction in surgical error. There are few studies studying functional outcomes post robotic-assisted UKA. The aim of this study was to compare the functional outcomes between robotic-assisted and conventional medial UKA.

**Material and Methods:**

A retrospective review was done of 159 patients; 110 patients underwent conventional UKA while 49 patients underwent robotic-assisted UKA. Outcome measures included the Oxford Knee Score (OKS), Knee Society Score (KSS), Visual Analogue Score (VAS) for pain, and range of motion (ROM) at three months, one-year and two years post-UKA.

**Results:**

Pre-operative patient demographics and outcome scores were not significantly different between both groups. ROM was significantly greater in the MAKO compared to the Oxford group at 3 months (p=0.039), 1 year (0.053) and 2 years (0.001) post-operation. While OKS, KSS and VAS scores improved for both groups, there were no significant differences in the final outcome measures. None of the patients experienced a mechanical failure, infection, or revision post-surgery. One patient each in the Oxford and MAKO group suffered a periprosthetic fracture.

**Conclusion:**

Both robotic-assisted MAKO UKA and conventional Oxford UKA showed good clinical outcomes. Robotic-assisted MAKO UKA had superior ROM outcomes compared to conventional Oxford UKA up to two years post-surgery.

## Introduction

Unicompartmental knee arthroplasty (UKA) was first introduced by Repicci *et al*^1^ in 1990s for unicompartmental knee osteoarthritis. Its significant advantages over total knee arthroplasty (TKA) include the restoration of knee kinematics^2^, minimising blood loss^3^, earlier mobilisation and shorter recovery time^4^. Despite the benefits of UKA, due to its need for precise alignment, positioning and soft tissue balancing, UKA failures and revision rates may be higher than that of TKA^5-9^. Studies have shown that UKA failures can be attributed to component malposition^10^, where even small errors in the coronal plane increases the risk of failure^5,7,8,11^.

To improve the accuracy of surgery, robotic assisted surgery has been introduced in recent years. Studies have suggested more accurate implant positioning^10,12-14^, improved alignment of the lower limb^15,16^ and soft tissue balancing^17^, as well as a reduction in surgical error as compared to conventional UKA^14,18,19^. While there are studies describing increased accuracy and comparable survivorship, there are only few studies studying functional outcomes between the two groups. The mobile bearing Oxford UKA has a more congruent bearing surfaces with a large contact area, generating less contact stresses^20^. The robotic UKA uses a fixed bearing prosthesis, which is theoretically less conforming and can lead to point loading^21^. Studies have compared conventional mobile bearing and fixed bearing UKA – however, there have been minimal studies comparing robotic assisted fixed bearing and conventional Oxford mobile bearing UKA.

The aim of this study was thus to compare the functional outcomes between robotic assisted fixed bearing and conventional mobile bearing Oxford UKA.

## Materials and Methods

**Fig. 1:**
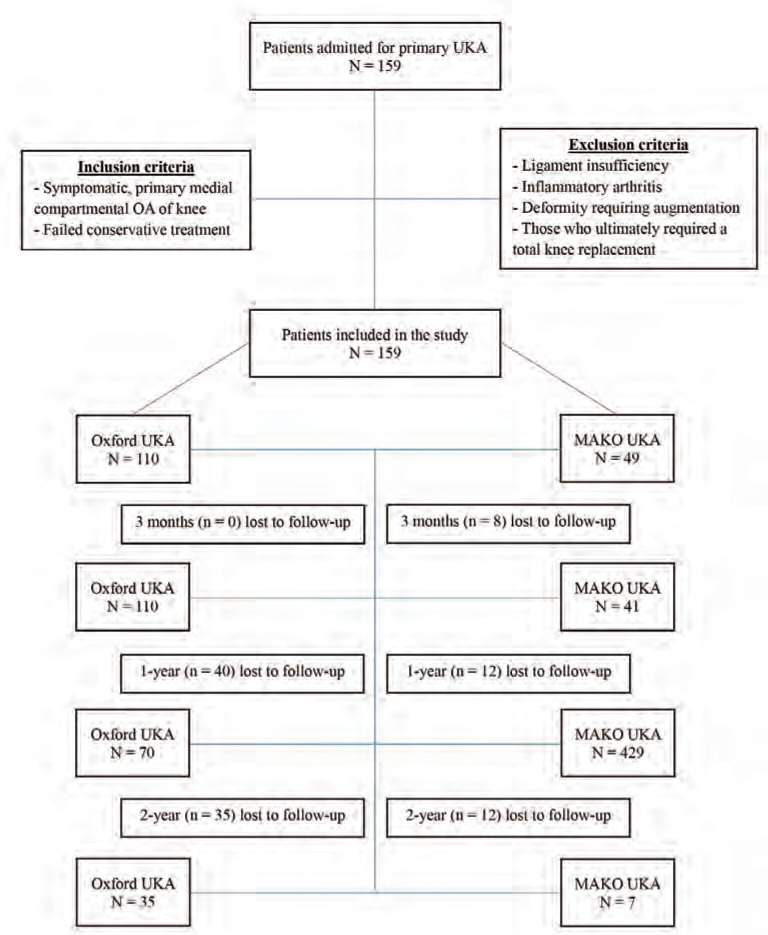
Selection process of patient records that were analysed.

A retrospective, single centre study was performed of 159 patients who underwent UKA for the treatment of medial compartment knee osteoarthritis (OA). UKA was performed with conventional instrumentation (n=110) or robotic technology (n=49), between September 2017 and December 2020 by a total of 6 surgeons. A paramedial quadriceps sparing approach was used for both groups. For patients undergoing conventional instrumentation, Oxford implants were used, while MAKO robotic system was used with RESTORIS ® MCK components. The inclusion criteria for patients for UKA included (1) radiographic evidence of medial unicompartmental OA and the absence of lateral compartment or patellofemoral compartment wear on the anterior-posterior, skyline and Rosenberg views, (2) clinical evidence of medial joint line tenderness, the absence of tenderness over the lateral joint line or patellofemoral region, flexion of minimally 90°, an intact anterior cruciate ligament, a fixed flexion deformity of less than 15° and a varus deformity of less than 10°. Exclusion criteria included ligament insufficiency, inflammatory arthritis, deformity regarding augmentation, and those who ultimately required a TKA (Fig. 1).

Institutional review board approval was attained by the authors’ affiliated institutions. Consent from patients was attained. Oxford implants: The Oxford III consists of a spherical articular surface with a fully congruent mobile meniscal bearing. Pre-operative templating was performed to select implant sizes. A paramedial quadriceps sparing approach was used, and conventional instrumentation done using extra and intramedullary cutting jigs. Uncemented or cemented implants were inset with the mobile bearing insert.

**Fig. 2:**
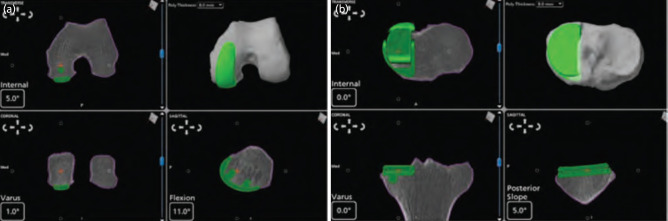
(a) Pre-operative planned femoral resection and implant positioning. (b) Pre-operative planned tibial resection and implant positioning.

Robotic assisted (MAKO): The RESTORIS® MCK is a cobalt chrome femoral component, fixed polyethylene bearing and a titanium tibial component. A pre-operative CT was performed to aid in the construction of a 3D model on the computer system. This allowed for planned femoral and tibial resections, and implant positioning on the 3D model (Fig. 2). A paramedial approach was again utilised. Markers were placed on the tibia and femur with stab incisions. Both tibial and femoral anatomical landmarks were recorded, mapping the surgical field data to the pre-operative CT. This allowed dynamic referencing. Soft tissue balancing was then carried out and recorded by the system. A high-speed burr was controlled by a tactile guidance system (visual, auditory and haptic feedback). Prior to each section of bone prepared, tibial and femoral checkpoints were verified. The tracker probe was placed to visualise accuracy and cuts onto the CT scan. The tibia and femoral surfaces were prepared, and implants cemented. At our institution, patients followed an identical post-operative rehabilitation program in a specialised arthroplasty ward with trained nurses and physiotherapists. Patients started immediate post-operative weight bearing, with necessary ambulatory aids as assessed by the physiotherapist. Range of motion and isometric exercises were also taught to the patient.

Patient reported outcomes were collected at three months, one year and two years post-operatively. Outcome measures collected included the Oxford Knee Score (OKS), Visual Analog pain score (VAS), Knee Society Score (KSS), and the range of motion (ROM). In addition, pre-operative demographics and outcome scores, intra-operative and hospital variables were also collected (Tables I and II).

**Table I:** Pre-operative demographics and outcome scores of patients undergoing unicompartmental knee arthroplasty.

	Total, n = (%)	Type of UKA MAKO, n = 49 (%)		Oxford, n = 110 (%)	p-value (CI)	Odds ratio
Body Mass Index, kg/m^2^, mean		27.6		28.1	0.590 (-1.17, 2.05)	
Age, years, mean		63.5		63.2	0.847 (-3.42, 2.81)	
Gender						
Male	62 (39.0)	15 (24.2)		47 (75.8)	0.163	1.40
Female	97 (61.0)	34 (35.1)		63 (64.9)		
Race						
Chinese	119 (74.8)	36 (30.3)		83 (69.7)	0.132	
Malay	19 (11.9)	3 (15.8)		16 (84.2)		
Indian	15 (9.4)	8 (53.3)		7 (46.7)		
Others	6 (3.8)	2 (33.3)		4 (66.7)		
Oxford Knee Score (OKS), mean		26.61		27.36	0.564 (-1.82, 3.32)	
Visual Analogue Pain Score (VAS), mean		5.63		6.06	0.223 (-0.26, 1.12)	
Knee Society Score (KSS), mean		52.55		48.72	0.102 (-8.43, 0.77)	
Range of motion (ROM), mean		115.1		111.0	0.145 (-9.69, 1.44)	

**Table II:** Intra-operative and hospital variables of patients undergoing unicompartmental knee arthroplasty.

	Total, n = (%)	Type of UKA		p-value (CI)	Odds ratio
		MAKO	Oxford		
Tourniquet Time, mean, (standard deviation), minutes		75.37 (40.36)	74.26 (18.95)	0.813 (-10.39, 8.17)	
Surgery Time, mean, (standard deviation), minutes		127.45 (36.72)	92.21 (19.55)	<0.001 (-43.82, -26.66)	
Length of Stay, mean, (standard deviation), days		3.94 (1.97)	4.32 (1.43)	0.182 (-0.18, 0.94)	
Blood loss, n = (%)					
<50 ml	53 (33.3)	13 (26.5)	40 (36.4)	0.023	
50 ml	40 (26.4)	18 (36.7)	24 (21.8)		
100-150 ml	39 (24.5)	15 (30.6)	24 (21.8)		
200 ml	20 (12.6)	1 (2.0)	19 (17.3)		
>300 ml	5 (3.1)	2 (4.1)	3 (2.7)		
Tranexamic acid use, n = (%)					
Yes	155 (97.5)	48 (31.0)	107 (69.0)	1.000	
No	4 (2.5)	1 (25.0)	3 (75.0)		1.346
Dexamethasone use, n = (%)					
Yes	84 (52.8)	42 (50.0)	42 (50.0)	<0.001	
No	75 (47.2)	7 (9.3)	68 (90.7)		9.714
Anaesthesia type, n = (%)					
Spinal	91 (57.2)	27 (29.7)	64 (70.3)	0.732	1.134
GA	68 (42.8)	22 (32.4)	46 (67.6)		
PCA use post-operatively, n = (%)					
Yes	30 (18.9)	4 (13.3)	26 (86.7)	0.027	
No	129 (81.1)	45 (34.9)	84 (65.1)		0.287
Local infiltration of analgesia (LIA)					
use, n = (%)					
Yes	152 (95.6)	48 (31.6)	104 (68.4)	0.439	
No	7 (4.4)	1 (14.3)	6 (85.7)		2.769

Statistical analyses were performed using SPSS software ver. 22.0 [IBM, Armonk, NY, USA]. A probability (p) value of less than 0.05 was considered statistically significant. Chisquare test was used to compare categorical variables. Normally distributed continuous variables were analysed using the Student’s T-test. ANOVA regression was used for multi-variate analysis.

## Results

A total of 159 patients were initially included in the study, of which 110 patients underwent conventional Oxford UKA and 49 underwent robotic assisted MAKO UKA. Four patients were lost to follow-up after 3 months, 56 after 1-year and 57 after 2 years (Fig. 1). Pre-operative demographics of BMI, age, gender, and race were not significantly different between both groups. There was also no significant difference in the pre-operative outcome scores between patients of both groups (Table I). Mean surgery time was found to be significantly shorter for patients who underwent Oxford UKA (p<0.001) (Table II).

ROM was significantly greater in the MAKO group compared to the Oxford group at 3 months (p=0.039), 1 year (0.053) and 2 years (0.001) post-operation. While OKS, KSS and VAS scores improved for both groups, there were no significant differences in the final patient reported outcome measures (PROM) (Tables III, IV, V). None of the patients experienced a mechanical failure, infection, or revision post-surgery. Two patients in the Oxford UKA group had deep vein thrombosis post-surgery, which was resolved with anticoagulation. One patient in the Oxford and MAKO group each suffered a periprosthetic fracture, of which the patient in the Oxford group was managed conservatively.

**Table III:** Three months outcome scores comparing robotic assisted and conventional unicompartmental knee arthroplasty.

	Total,	Type of		UKA	p-value (CI)	Odds ratio
	n = 155 (%)	MAKO, n = 41		Oxford, n = 110		
Oxford Knee Score (OKS), mean		40.22		38.49	0.136 (-4.01, 0.55)	
Visual Analogue Pain Score (VAS), mean		1.49		1.23	0.445 (-0.93, 0.41)	
Knee Society Score (KSS), mean		83.15		82.73	0.861 (-5.08, 4.25)	
Range of motion (ROM), mean		121.0		115.3	0.039 (-11.05, -0.30)	

**Table IV:** One-year outcome scores comparing robotic assisted and conventional unicompartmental knee arthroplasty.

	Total, n = 99 (%)	Type of UKA		p-value (CI)	Odds ratio
		MAKO, n = 29	Oxford, n = 70		
Oxford Knee Score (OKS), mean		42.86	43.11	0.811 (-1.83, 2.34)	
Visual Analogue Pain Score (VAS), mean		1.10	0.81	0.474 (-1.09, 0.51)	
Knee Society Score (KSS), mean		85.36	85.53	0.933 (-3.86, 4.20)	
Range of motion (ROM), mean		127.07	122.61	0.053 (-8.97, 0.06)	

**Table V:** Two-year outcome scores comparing robotic assisted and conventional unicompartmental knee arthroplasty.

	Total, n = 42 (%)	Type of UKA		p-value (CI)	Odds ratio
		MAKO, n = 7	Oxford, n = 35		
Oxford Knee Score (OKS), mean		44.14	43.57	0.701 (-3.56, 2.42)	
Visual Analogue Pain Score (VAS), mean		0.86	0.63	0.762 (-1.74, 1.29)	
Knee Society Score (KSS), mean		83.71	83.83	0.977 (-7.95, 8.17)	
Range of motion (ROM), mean		132.57	121.57	0.001 (-17.52, -4.48)	

## Discussion

Robotic assisted UKA has shown improvements in the accuracy of implant positioning^10,12-14^, alignment of the lower limb^15,16^, as well as a reduction in surgical error as compared to conventional UKA^17-19^. There have been fewer studies on the impact on post-operative PROM comparing robotic assisted fixed bearing and conventional mobile bearing UKA. Our study showed that robotic assisted MAKO UKA and Oxford UKA have improved KSS, OKS and VAS scores three months, one year and two years post-surgery. The one-year improvement in mean OKS scores were 16.25 and 15.75 for the MAKO and Oxford groups, respectively. This exceeds the minimally important clinical difference (MICD) for this PROM(a) ^22^. In addition, the one-year improvement in mean KSS scores were 32.81 and 36.81 for the MAKO and Oxford groups, respectively. This similarly exceeds the MCID for this PROM^23^.

While there was an improvement in PROMs in both groups, there was no significant difference in the final KSS and OKS scores at three months, one year and two years post-surgery (Tables III, IV, V). A prospective study by Blyth *et al* studied 139 patients undergoing UKA^24^, with patients randomised to either conventional or robotic assisted UKA. The follow-up study was published again by Gilmour *et al* studying the two-year outcome scores between both groups^25^. The authors found that while there were significantly better KSS scores for patients who underwent MAKO UKA after three months, this difference in mean KSS narrowed after one year, with no difference in clinical outcomes two years post-surgery. A study done by Crizer *et al* also similarly showed that while there was earlier functional recovery in robotic assisted patients up to six months post-surgery, there was no difference in one-year KSS scores between both groups of robotic and conventional UKA^26^. As such, this shows that though immediate short-term post-surgery outcomes may be better for patients undergoing robotic assisted UKA, these differences equilibrate after one- and two-years post-surgery. Our study also showed that while VAS scores improved in both the MAKO and Oxford UKA groups, there was no significant difference in the final VAS scores. Similarly, Crizer *et al*^26^ reported no difference between both groups with respect to the decrease in VAS pain scores. However, in the prospective study done and published by Blyth *et al*^24^ and Gilmour *et al*^25^, there was lower early pain and a higher percentage of patients ‘pain free’ at two years in the robotic group. A prospective cohort study of 146 patients by Kayani *et al*^27^, showed that robotic assisted UKA was associated with reduced post-operative pain and decreased opiate analgesic requirements compared to conventional. We suggest that other factors such as comparative pre-operative pain levels, change in pain levels pre- and post-surgery and, perioperative analgesia regime between both groups should also be studied. Three months, one year and two-year ROM was found to be significantly improved for MAKO assisted fixed bearing UKA compared to conventional mobile bearing Oxford in our study (p=0.039, p=0.053 and p=0.001, respectively). Mobile bearing prostheses offers more congruent bearing surfaces with a large contact area^20^, while the fixed bearing prosthesis has a flat tibial articular surface and is less conforming during flexion^21^. This theoretically means that the mobile bearing Oxford should confer a better ROM. However, studies have shown no difference in mean maximal ROM or functional status between the mobile and fixed bearing designs of knee arthroplasty^28-30^. Gilmour *et al*^25^ had comparable findings to our study, with patients in the robotic-arm assisted group achieving a significantly greater improvement in ROM after two years. As such, this may imply that a robotic assisted fixed bearing device confers better ROM than a conventional Oxford, despite the Oxford’s mobile bearing.

Our study has several limitations. Firstly, as it is a retrospective study, there is a potential for selection bias. However, statistical analysis showed no significant differences in patient demographics, pre-operative PROMs and ROM between both groups – alleviating such a bias. Secondly, pre-operative activity level was not studied for our patients, which may be a potential confounder. A sub-analysis of patients with a better pre-operative activity level may provide greater insight into the benefit on their functional outcomes. In addition, a further analysis of our patient population to evaluate the survival rate of medial UKA in comparison to the available literature can be done. Heaps *et al* quoted a five- and ten-year pooled survival rate of 95.3% and 91.3%, respectively for medial UKA survival rates^31^. Also, there were a number of patients which were lost to follow-up, which decreased our sample size during follow-up data collection.

The effect of robotic assisted UKA compared to conventional UKA on component alignment was also recorded in our patient population, but not included in this study. It is currently being evaluated in a follow-up study.

## Conclusion

In conclusion, our study demonstrates that at three months, one-year and two years post-operatively, both robotic assisted MAKO UKA and conventional Oxford UKA showed good clinical outcomes, with improvements in KSS, OKS and VAS scores. Robotic-assisted MAKO UKA had superior ROM outcomes compared to conventional Oxford UKA up to two years post-surgery. These results are promising and suggest that further mid- and long-term studies can better assess whether robotic-assisted UKA can provide improved outcomes in the long-term.
